# Context expectation influences the gait pattern biomechanics

**DOI:** 10.1038/s41598-023-32665-7

**Published:** 2023-04-06

**Authors:** Tommaso Ciceri, Giorgia Malerba, Alice Gatti, Eleonora Diella, Denis Peruzzo, Emilia Biffi, Luca Casartelli

**Affiliations:** 1 Department of Information Engineering, University of Padova, Padua, PD Italy; 2 Neuroimaging Lab, Scientific Institute IRCCS E. Medea, Bosisio Parini, LC Italy; 3 Bioengineering Lab, Scientific Institute IRCCS E. Medea, Bosisio Parini, LC Italy; 4 Department of Electronics, Information and Bioengineering, Politecnico di Milano, Milan, MI Italy; 5 Theoretical and Cognitive Neuroscience Unit, Scientific Institute IRCCS E. Medea, Bosisio Parini, LC Italy

**Keywords:** Cognitive neuroscience, Motor control

## Abstract

Beyond classical aspects related to locomotion (*bio*mechanics), it has been hypothesized that walking pattern is influenced by a combination of distinct computations including online sensory/perceptual sampling and the processing of expectations (*neuro*mechanics). Here, we aimed to explore the potential impact of contrasting scenarios (“risky and potentially dangerous” scenario; “safe and comfortable” scenario) on walking pattern in a group of healthy young adults. Firstly, and consistently with previous literature, we confirmed that the scenario influences gait pattern when it is recalled concurrently to participants’ walking activity (motor interference). More intriguingly, our main result showed that participants’ gait pattern is also influenced by the contextual scenario when it is evoked only before the start of walking activity (motor expectation). This condition was designed to test the impact of expectations (risky scenario vs. safe scenario) on gait pattern, and the stimulation that preceded walking activity served as prior. Noteworthy, we combined statistical and machine learning (Support-Vector Machine classifier) approaches to stratify distinct levels of analyses that explored the multi-facets architecture of walking. In a nutshell, our combined statistical and machine learning analyses converge in suggesting that *walking before steps* is not just a paradox.

## Introduction

Considering that healthy adults are generally able to adjust their gait pattern continuously and flexibly to accommodate environmental and contextual requirements, bipedal gait is often taken for granted by most people. Nevertheless, gait should be considered a challenging activity from a biomechanical perspective. Seminal studies reported that the vertical projection of the centre of mass is outside the base of support for approximately 80% of the gait cycle, de facto involving continuous falling motion in which the base of support is relocated step by step^1,2^. Recent advances in neuroscience strongly link *bio*mechanics and *neuro*mechanics of walking, sparking intriguing and promising insights into the understanding of non-motor computations driving walking activity^3^. This picture leads to strong theoretical and clinical concerns whenever walking activity is not fluid, efficient or stable as often reported following acquired (e.g., stroke; traumatic brain injury), congenital (e.g., cerebellar agenesis or malformations) or neurodegenerative (e.g., Parkinson disease) conditions^4–6^.

Dominant approach tended to use handy classifications distinguishing among lower-level (musculoskeletal or peripheral nervous system disturbances), middle-level (e.g., basal ganglia or cerebellar related disturbances) and higher-level (e.g., cortico-cortical or cortico-subcortical connectivity disturbances) gait disorders^7^. For decades, this classical clinical framework was considered the more reliable one, and it was largely employed to address early prodromic gait signs in dementia according to the so-called “last in-first out” approach. In neurodegenerative conditions such as Alzheimer’s disease or frontotemporal dementia, this approach was hypothesized to be critical considering the strict link between gait disturbances and cognitive functioning decline reported in these clinical conditions^8^. The “last in-first out” approach assumes that the progression of degeneration follows the course of neurotypical maturation in reverse order (a phenomenon known as “retrogenesis”). In other words, this approach assumes that brain circuits most vulnerable to early neurodegeneration are the ones that mature late in ontogeny, whereas the ontogenetically more ancient nodes are the ones that are preserved for more time^9^. Thus, signs of gait pattern disturbances in older people may be considered as red flags for neurodegenerative conditions, in turn suggesting clinical monitoring of motor and non-motor (i.e., neurocognitive) functioning^10^. Despite its clinical relevance, we suspect this three-level approach to gait disturbances lacks additional subtler aspects related to the *neuro*mechanics of gait. A paradigm-shift is necessary.

We conjectured that walking pattern is influenced by a combination of online sensory and perceptual stimulation, prior knowledge, and current expectations. Each component is weighted flexibly and adaptively in healthy individuals, and their combination assumes a central role in driving walking pattern. This would lead to theorize that walking activity is neither a monolithic process nor a rigid or inflexible phenomenon, whereas walking is characterized by highly flexible, multi-facets and layered architecture. Accordingly, healthy walkers not only would combine visual scene processing (e.g., a mountain pathway) with online sensory parsing (e.g., the proprioceptive feedback of asphalt or dirt road)^11,12^. Healthy walkers would combine also their prior knowledge concerning that specific terrain (e.g., this mountain trail is easy when it is dry, but it is dangerous when it is damp), and current expectation (e.g., this slope is shady in winter until 11 a.m.; yesterday it rained; now it is 9 a.m., it will be damp and I have to be prudent). It exemplifies our hypothesis concerning distinct computations contributing to regulate walking activity. Among them, the computations that refer to the individual evaluation of the sensory/perceptual world (in terms of expectations resulting from the active and dynamic collection of information) would play a critical role^13–16^. Although such a view appears theoretically convincing, there is scarce experimental evidence directly supporting it. In addition, they mostly relied on the visual or proprioceptive domain. Here, we try to fill this gap by testing the auditory one.

We investigated the impact on walking patterns of two contrasting pictures evoking a “risky and potentially dangerous” scenario (i.e., seaside during a flashing autumnal storm), and a “safe and comfortable” scenario (i.e., a sunlit and bright summer day). These scenarios were depicted through ad hoc audio-descriptions provided by a professional actor, and they were associated with correspondent audios (stormy-seaside audio and sunny-seaside audio, respectively). First, we aimed to explore the potential impact of risky/safe audios (as proxies of the risky and safe audio-description, respectively) concurrent with walking activity, a condition we referred to as “Motor Interference” (MI). This should represent a sort of internal control condition, being already reported in the literature comparable interference effects on motor outputs. Notably, it was reported that walking speed is affected when participants are provided with sounds simulating steps on different terrains (e.g., snow) compared to the one they are walking upon^17,18^. Our second and principal aim was to explore the potential impact of risky/safe audios employed as priors just before the starting of gait activity, a condition we referred to as “Motor Expectation” (ME). Indeed, in this condition walking activity is not directly interfered by any audio (and/or visual) stimulation, whereas it is influenced only by the expectation of a risky/safe scenario evoked by the (auditory) prior. Noteworthy, participants were not aware of any aim of the study. They were just asked to listen attentively the audio-description (risky/safe scenario) or the correspondent audio (risky/safe), and to walk in a self-paced modality. Thus, gait pattern modifications within and between conditions should be attributed to an implicit impact (i.e., a non-explicitly requested, and not even insinuated impact) of the concurrent or anticipatory stimulation (MI or ME condition, respectively). We also collected participants’ feedback at the end of the experiment, notably focusing on the individual post-session awareness concerning the putative impact of distinct scenarios on their own walking pattern. In sum, our hypothesis was that such an experimental setting could reliably show the effect of both concurrent and anticipatory stimulation on gait pattern. If—and eventually how much—these effects are similar/different, will be clarified by results.

## Methods

### Experimental design

**Figure 1:**
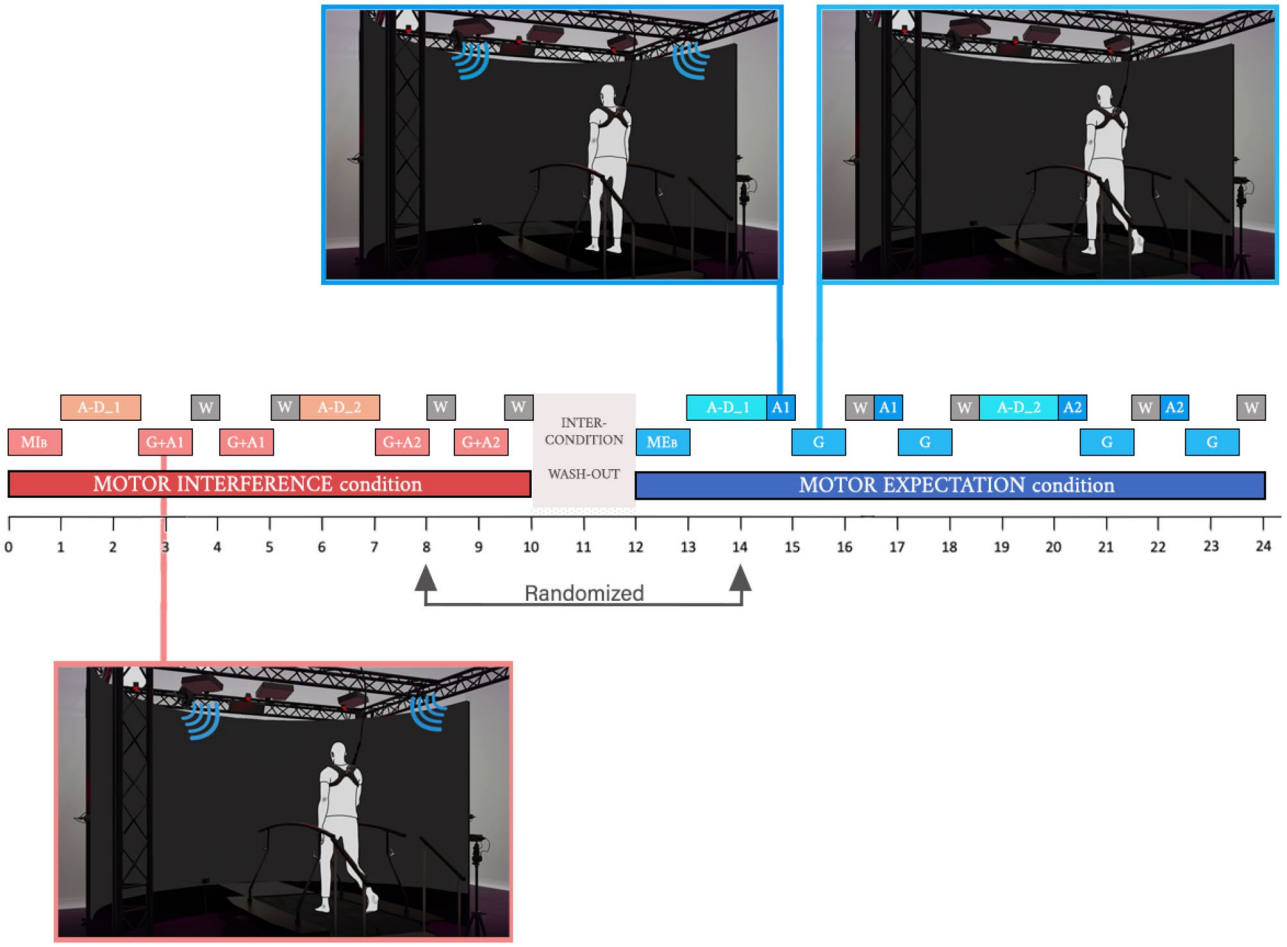
Graphical representation of the experimental procedure. The two distinct conditions (MI, ME) are separated by a wash-out period. Timeline (in minutes) is reported. The term “randomized” in the figure means that the order of presentation of MI and ME condition was counter-balanced across participants. In addition, also scenarios (risky, safe) were counter-balanced across participants. This means that the A-D_1 and A-D_2, G + A1 and G + A2, A1 and A2 are used in the figure to refer alternatively to the “risky (safe)” or “safe (risky)” scenario. MI: Motor Interference condition; ME: Motor Expectation condition; MI_B_: Motor Interference baseline; ME_B_: Motor Expectation baseline; A-D_1: Audio-description (risky/safe); A-D_2: Audio-description (safe/risky); G + A1: Gait + Audio (risky/safe); G + A2: Gait + Audio (safe/risky); A1: Audio (risky/safe); A2: Audio (safe/risky); G: Gait; W: Wash-out.

We aimed to elicit two contrasting experimental scenarios in each participant. Notably one of them should recall a “risky and potentially dangerous” scenario (i.e., seaside during a flashing autumnal storm), whereas the other one should recall a “safe and comfortable” scenario (i.e., a sunlit and bright summer day). We elicited them with specific ad hoc audio-description provided by an Italian professional actor that read two brief contrasting stories (hereafter, “audio-description risky” and “audio-description safe”; for the exact text employed, see Supplementary Information). We chose to use the voice of a single actor to avoid potential biases due to individual vocal characteristics, and they were originally provided in Italian being all participants Italian native speakers. Both audio-descriptions last 90-s, and they were associated with the correspondent audio (stormy seaside-audio and sunny seaside-audio, respectively). This should promote an associative link between the audio-descriptions and the correspondent audios (hereafter, “audio risky” and “audio safe”). Thus, audio risky corresponded to a stormy-seaside audio (the same one used as background sound during the audio-description of the scenario “risky”), and the audio safe corresponded to a sunny-seaside audio (the same one used as background sound during the audio-description of the scenario “safe”). Noteworthy, no visual stimulation was provided, and participants simply stayed in a dimly illuminated room (just for safety reason) (see, Fig. 1).

Two experimental conditions were set: “Motor Interference” condition (MI), and “Motor Expectation” condition (ME). MI aimed to explore putative gait pattern modifications due to auditory stimulation (i.e., audio risky and audio safe) provided simultaneously to the gait pattern recording. This should be considered a sort of internal control condition to test the audio-motor interference effect on gait pattern. In contrast, ME condition provided the audios (i.e., audio risky and audio safe) before the starting of gait pattern recording, when participants were not requested to walk (participants were simply requested to listen attentively the audios). This should test the main hypothesis of the present study concerning the putative effect of auditory stimulations used as priors on gait pattern. Counter-balancing of conditions and scenarios is described in the Participants section.

Before starting each condition (MI or ME), a 1-min walking period of baseline (B) was performed (hereafter MI_B_ or ME_B_, respectively. For all abbreviations of this section, see also Fig. 1). In the MI condition, after the baseline (MI_B_) the audio-description of the first scenario (hereafter, A-D_1) was presented. Just after participants were requested to walk for 1-min while the correspondent audio (audio1, hereafter A1) was simultaneously proposed in the background (gait + audio1 phase, hereafter G + A1). A 30-s wash-out (W) period followed, in which participants were free to distract themselves and no requests were provided. After it, a second gait + audio phase (G + A1) and a further wash-out period were proposed. In turn, the same sequence with the alternative scenario was proposed (i.e., A-D_2; G + A2; W; G + A2; W). Considering the ME condition, the 1-min baseline (ME_B_) was followed by the audio-description of the first scenario (A-D_1). Then, a 30-s audio (A1) was proposed in the background to participants while they were not requested walking (they were simply asked to listen attentively the audio). In turn, “pure” walking activity (without any background audio) was performed for 1-min by participants (G). After walking, a 30-s wash-out period (W) and a second audio plus gait plus wash-out combination was repeated (A1; G; W). In turn, the same sequence with the alternative scenario was proposed (A-D_2; A2; G; W; A2; G; W). Noteworthy, in the ME condition the audios associated to the scenarios (A1/A2) were presented before the gait activity phase. This is hypothesized to work as a prior that impacts on gait pattern. A 2-min period was set as wash-out between conditions. This should minimize potential contamination cross-effects between conditions.

For sake of simplicity, Fig. 1 describes the experimental procedure starting with the MI condition, and using A-D_1 and A-D_2, G + A1 and G + A2, A1 and A2 for referring alternatively to the “risky (safe)” or “safe (risky)” scenario (e.g., for participant Tom, A-D_1 corresponds to the audio-description risky, A1 to the audio-risky, A-D_2 to the audio-description safe, A2 to the audio-safe. For participant Jerry, A-D_1 corresponds to the audio-description safe, A1 to the audio-safe, A-D_2 to the audio-description risky, A2 to the audio-risky).

### Experimental setup

We run the experiment in the Gait Real time Analysis Interactive Lab (GRAIL, Motek, the Netherlands), an immersive virtual reality device equipped with a two degrees of freedom motion frame, integrated force plates (16 channels, sample frequency 1000 Hz), a motion-capture system (10 optoelectronic cameras, sample frequency 100 Hz), a Dolby surround system, a dual-belt treadmill, and a 180° cylindrical projection screen^19,20^. To assure safety, two lateral handrails and one harness are provided. Among multiple features of the system, our current experiment specifically benefited from the possibility to record multistep data describing spatio-temporal parameters, kinetics, and kinematics of the human gait. We also benefited from the possibility to use the self-paced modality (i.e., participants walked at their own favorable walking speed), and this guaranteed an individualized walking pattern for each participant. For the current experiment, the system also provided distinct auditory stimulations to participants.

A new ad hoc application (named “Sound”©2021, IRCCS Medea) was designed by using the D-flow, the software that oversees the relationship among the participant, the treadmill, and distinct stimulations. D-flow controls the hardware devices (i.e., treadmill, audio system, motion capture system in the current study) via the concept of “modules”. These modules communicate reciprocally, and they can be activated/deactivated according to specific needs of the experimental design^21^. Operationally, the experimenter was requested to activate specific “buttons” according to well-standardized written instructions. Specifically, one button activated the gait acquisition in self-paced modality (corresponding to MI_B_, ME_B_, and G in Fig. 1). Two buttons activated the two distinct audio-descriptions (audio-description risky and audio-description safe, respectively; corresponding to A-D_1 and A-D_2 in Fig. 1). Two buttons permitted the treadmill activation in self-speed mode together with the recording of gait patterns combined with, respectively, the playing of audio risky and audio safe (corresponding to G + A1 and G + A2 in Fig. 1). Other two buttons allowed to activate the audio risky and audio safe (corresponding to A1 and A2 in Fig. 1). Finally, one button activated a counter for the wash-out periods (corresponding to W in Fig. 1).

### Experimental protocol

The GRAIL system allows motion data recording and processing in real time by means of the Human Body Model II (HBM-II^22^). Accordingly, 26 markers were placed on specific anatomical landmarks of the body. They were used to recreate the mechanical model of the two legs and of the trunk. Participants were requested to wear appropriate clothing (i.e., sports underwear) that did not prevent the reliable acquisition of movements by covering the markers during the gait activity.

Each participant performed a 10-min familiarization phase in which she/he was requested to repeatedly start/stop gradually her/his walking activity over the treadmill, and to walk in self-paced mode. This phase ensured that the participant was comfortable enough while walking on the treadmill and his/her gait was stable. Then, participants were driven in all distinct steps of the experimental procedure with easy-to-understand and standardized verbal instructions provided by the experimenter. This was provided to avoid the accidental use of any terms that may bias participants’ walking activity (all experimental steps in which participants were requested to walk, they freely walked at her/his own comfortable pace, i.e., self-paced modality). For safety and technical reasons, we also set the maximum gait speed each participant could reach (individualized on the last two minutes of her/his familiarization phase, i.e., comfortable speed increased by 44%). Concerning the audio stimulation, volume was set equal for all participants briefly piloting distinct options before the beginning of the recruitment (we simply need that the audio-descriptions and audios were clearly audible net of the GRAIL system mechanic noise). At the end of the experimental session, through ad hoc standardized written questions, we briefly asked feedback from participants concerning their awareness of the putative impact of distinct scenarios on their own walking patterns. This was a very simple and ecological way to monitor the potential differences in the individual *explicit* awareness during concomitant (MI) or preceding (ME) audio stimulation.

### Participants

32 healthy, Italian native speaker young adults (16 females, age 27.0 ± 3.4 years; 16 males, age 27.1 ± 5.2 years) participated to this experiment. Participants were recruited following specific selection criteria: they did not have neurological or neuropsychiatric diagnosis, and they did not have any clear sign of neurological, musculoskeletal, or other temporary (e.g., backache) clinical condition that could have affected their postural control. Noteworthy, participants were not aware in advance neither of the purpose of the study, nor of the experimental procedure.

We counter-balanced both conditions (MI, ME) and scenarios (risky, safe) across participants, keeping into account the gender variable. This should minimize potential order-effects or implicit biases due to the presentation of one specific scenario/condition first respect to the other.

The entire experimental procedure was approved by the Ethics Committee of the Scientific Institute IRCCS E. Medea (Bosisio Parini, Italy). Written informed consent was obtained from each participant. The study was conducted according to the principles expressed in the Declaration of Helsinki.

### Data analysis

GRAIL data were pre-processed by using the Gait Offline Analysis Tool (GOAT). GOAT synchronizes and displays 3D motion capture and force plates data together with video recordings, allowing a comprehensive analysis. It also performs real-time filtering of the GRAIL data with a low-pass 2nd order Butterworth filter, with a cut-off frequency equal to 6 Hz. The GOAT software features built-in gait event detection algorithms and standardized step selection, providing optimal processing for all treadmill-based gait data. GOAT normalizes the steps on 100 samples, returning a single value for the space–time parameters and time series for kinetic and kinematic parameters. After a first qualitative and screening investigation, the first 10 steps of both conditions recorded in the acquisition were not considered and eliminated, since they concerned the phase in which participants set their self-paced speed.

Spatio-temporal parameters (e.g., walking speed, stance duration, stride length, and step width), kinetic and kinematic parameters (related to pelvis, hip, knee, and ankle) were computed for each step with an ad hoc algorithm in MATLAB (for a similar approach, see Ref.^23^). For each participant, mean and standard deviation of distinct values (e.g., min, max, ROM, time, etc.) were extracted considering all the steps. Being involved healthy participants, we assumed no differences between the two sides of the body. Thus, for each parameter and each value, we averaged data of the right and left leg. As described in the Experimental Design section (Fig. 1), each condition has two “repetitions” (e.g., “A-D_1; G + A1; W; G + A1; W” for MI, and “A-D_2; A2; G; W; A2; G; W” for ME; we use the term repetition for “G + A1”—“G + A1” in MI, and “G”—“G” in ME). Repetitions were averaged within each condition (when both of them were available; 8/320 repetitions missing).

### Statistical analysis

The software R was used to conduct the statistical analysis of the pre-processed data. The non-normality of the group of participants was first evaluated by the Shapiro–Wilk test. Therefore, data were presented as median and interquartile range (IQR, computed as the 75th percentile minus the 25th percentile). The paired non-parametric Friedman Test, and the Wilcoxon Test were performed to explore potential significant results in the inter-scenarios and inter-conditions analyses.

The inter-scenarios analysis aimed to compare walking in the baseline period (B), in the risky scenario (R), and in the safe scenario (S) within participants for each experimental condition. The Friedman test was first performed among the baseline (B) and the scenarios (R, S) (statistical significance level, p < 0.05). The paired non-parametric Wilcoxon test with Bonferroni correction was applied as post-hoc analysis on the significant results of the Friedman test, considering a p-value corrected (p = 0.05/3). The new value of the significance level was set to p < 0.016.

The inter-conditions analysis was conducted on the participants’ walking activity between the two experimental conditions (MI, ME), both for the risky and safe scenario detrended on the respective baseline. The detrend was performed by subtracting the value at baseline to the value in the risky/safe scenario for each parameter (Eqs. 1–4):

1 $$\Delta M{I}_{S}=M{I}_{S}-{MI}_{B},$$

2 $$\Delta M{I}_{R}=M{I}_{R}-{MI}_{B},$$

3 $$\Delta M{E}_{S}=M{E}_{S}-{ME}_{B},$$

4 $$\Delta M{E}_{R}=M{E}_{R}-{ME}_{B}.$$

Where MI_S_ is the Motor Interference safe; MI_R_ is the Motor Interference risky; ME_S_ is the Motor Expectation safe; ME_R_ is the Motor Expectation risky; MI_B_ is the Motor Interference baseline; ME_B_ is the Motor Expectation baseline.

To investigate if there were statistically significant differences among the parameters in the two conditions, the paired non-parametric Wilcoxon test was performed between $\Delta M{I}_{S}$ and $\Delta M{E}_{S}$ and between $\Delta M{I}_{R}$ and $\Delta M{E}_{R}$. The significance level was established at p < 0.05. To sake of simplicity, hereafter we use MI_S_, MI_R_, ME_S_, ME_R_ to refer to the detrended values.

### Machine learning analysis

A machine learning-based analysis was performed on the pre-processed data extracted from the GRAIL system by using the GOAT using ad hoc MATLAB algorithms. This aimed to explore the possibility that the scenario (risky vs. safe) can be decoded from our set of parameters, and eventually to investigate which type of measure (spatio-temporal, kinetic, or kinematic) better discriminated the scenarios. Hereafter, we use the term “pooled” for referring to the analyses using all measures together (spatio-temporal + kinetic + kinematic), whereas we specifically refer to a subset (spatio-temporal or kinetic or kinematic) when the analyses consider one of them. This separation is not data-driven, but decided a-priori on the basis of the nature of the collected measures.

We used a Support-Vector Machine (SVM) classifier with a linear kernel combined with a preliminary principal component analysis (PCA) to reduce data dimensionality. We applied an 8-fold cross-validation procedure to avoid overfitting, and we assessed the prediction performances on an independent dataset^24^. The dataset was split into 8-folds containing 4 participants each. Participants were randomly assigned to a fold with the only constraint to preserve gender distribution across the different folds (i.e., each fold contains 2 males and 2 females). At each iteration, 6-folds (24 participants) were used as training set, 1-fold (4 participants) as validation set and 1-fold (4 participants) as test set. Folds were cycled among sets so that all of them were once used as test set. Preliminary operations, such as dimensionality reduction through PCA and value range standardization, were computed on the training set, and applied also to both the validation and test sets. The number of components retained with the PCA analysis was set to explain 95% of the variance of the original data^25^. The validation set was used in the classifier parameter optimization, it means that at each folding iteration the best error penalty parameter (C) was estimated with a grid search approach (ranging from [10^–5^ to 10^5^]). Classification performances were evaluated on the test set and reported in terms of accuracy, area under the receiver operating characteristic (ROC) curve (AUC), and F1-score. Finally, the significance of each performance index was computed using a permutation test with 10,000 permutations of the participant labels. We considered a performance index significantly larger than “the chance” (i.e., 50%) with a threshold set to p < 0.05. We performed four different classifications on the basis of the parameters employed to predict the scenario (i.e., pooled; spatio-temporal; kinetic; kinematic). In the first classification, pooled GRAIL derived parameters were concatenated, and then provided to the classifier. In the other ones, the parameters were grouped into three specific subsets (spatio-temporal; kinetic; kinematic).

## Results

### Statistical analysis

Our results convergently underlined the central role of the risky scenario in eliciting gait pattern modifications, whereas the safe scenario had only a marginal role (resulting as barely comparable to the baseline). Although we did not design the experiment assuming it, this is not surprising considering the current sample of healthy young adult participants. Indeed, we conjectured that also the safe scenario would assume more relevance in case of testing—for example—clinical populations with limitations in gait proficiency, a further step we plan to implement in the future. Thus, in the main text we follow a “risky-scenario-driven” presentation of data. This should promote a clearer picture of our results without disorienting the reader with marginal effects that can globally be considered negligible. However, for sake of completeness all data are reported in the Supplementary Information section.

The “risky-scenario-driven” results of the inter-scenarios analysis are reported in Table 1. Specifically, it reports the parameters that have significant results at the Friedman Test (p < 0.05), and having significant results at the post hoc test between the risky scenario versus the baseline (BR) as well as the risky scenario versus the safe one (SR) (Wilcoxon Test with Bonferroni correction, with p < 0.016) at least in one condition (MI and/or ME). Accordingly, Table 2 reports the inter-conditions analysis considering the same subset of parameters. As sketched above, parameters with a significant Friedman test in the inter-scenarios analysis but not satisfying the “risky-scenario-driven” approach requirements (i.e., they have not BR as well as SR significant differences in MI and/or ME, whereas they have at least one significant value among BR/BS/SR), are reported for sake of completeness in the inter-scenarios supplementary table (Supplementary Table S1), and in the inter-conditions supplementary table (Supplementary Table S2).

**Table 1:**
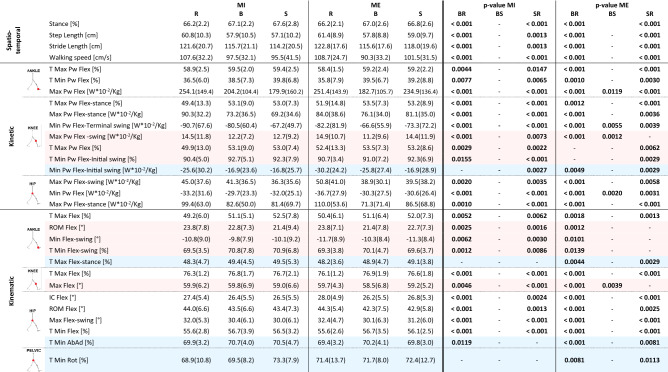
Results of the inter-scenarios analysis showing statistically significant results at the Friedman Test (p < 0.05), and having significant results at the post hoc test between the risky scenario versus the baseline (BR) as well as the risky scenario versus the safe one (SR) (Wilcoxon Test with Bonferroni correction, with p < 0.016) at least in one condition (MI and/or ME) are presented (“risky-scenario-driven” approach). Median and IQR values are reported. To improve readability, parameters having significant differences in both conditions (MI and ME) are reported on white background. Parameters having significant differences only in the MI condition are reported on pink background. Parameters having significant differences only in the ME condition are reported on light blue background. *MI:* Motor Interference condition, *ME:* Motor Expectation condition, *B:* Baseline, *R:* Risky scenario, *S:* Safe scenario, *BR:* Baseline vs. Risky scenario, *SR:* Safe scenario vs. Risky scenario, *BS:* Baseline vs. Safe scenario.

**Table 2:**
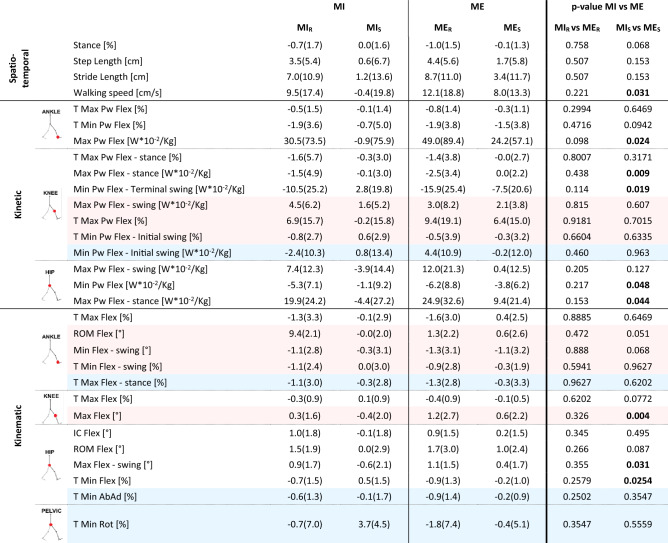
Results of the inter-conditions analysis considering the risky and safe scenarios detrended with respect to the baseline are presented. All parameters described in Table 1 are included (that is all parameters satisfying the “risky-scenario-driven” approach; i.e., significant differences in BR as well as in SR in at least one condition). Median and IQR values of the detrended parameters are reported (see Eqs. 1–4 in the Data Analysis section). P-values refer to the Wilcoxon test between MI and ME (i.e., MI_R_ vs. ME_R_; MI_S_ vs. ME_S_). Statistically significant values are in bold. To improve readability, colors conventions correspond to the ones in Table 1 (i.e., parameters having significant differences in BR as well as SR in both MI and ME condition are reported on white background; parameters having significant in BR as well as SR only in the MI condition are reported on pink background; parameters having significant differences in BR as well as in SR only in the ME condition are reported on light blue background). *MI:* Motor Interference condition, *ME:* Motor Expectation condition, *MI*_*S*_ : Motor Interference Safe (detrended), *MI*_*R*_ : Motor Interference Risky (detrended); *ME*_*S*_ : Motor Expectation Safe (detrended), *ME*_*R*_ : Motor Expectation Risky (detrended).

Focusing on the inter-scenarios analysis, most parameters showed a statistically significant difference in both conditions (MI and ME) between the risky scenario and the baseline (BR), as well as between the risky scenario and the safe one (SR) (Table 1, parameters with white background). These parameters highlighted changes in spatio-temporal features of the gait (i.e., the percentage of stance over the whole gait cycle, the step and stride length, and the walking speed), in its kinetics at the ankle (i.e., the time of maximum and minimum peaks of power and the maximum peak of power), at the knee (i.e., the time of maximum peak of power, the maximum peak of power in stance and the minimum peak of power in terminal swing), and at the hip (i.e., the maximum peaks of power in stance and in swing and the minimum peak of power), and in gait kinematics considering the ankle (i.e., time of maximum dorsiflexion), the knee (i.e., time of maximum flexion) and the hip (i.e., the time of minimum flexion, flexion at initial contact, ROM of hip flexion and maximum peak of flexion in swing). Specifically, the risky scenario was characterized by peaks with increased amplitude and with an early occurrence with respect to the baseline and the safe scenario. Interestingly, the inter-conditions analysis indicates that all these parameters did not differ between MI and ME condition in the risky scenario (Table 2, parameters with white background).

In addition, seven parameters had statistically significant differences in BR and SR but only in MI condition (Table 1, parameters with pink background). These parameters are the time of maximum peak of power at the knee, the time of the minimum power at the knee during the initial swing, the maximum peak of power in swing at the knee, the minimum ankle flexion in swing, the ROM of the ankle and the time of the minimum flexion of the ankle, and the maximum flexion of the knee. The inter-conditions analysis showed no statistically significant differences in the risky scenario (Table 2, parameters with pink background).

Finally, four parameters had a similar behavior but only in ME condition (Table 1, parameters with light blue background). They are the minimum peak of power of the knee in initial swing, the time of maximum dorsiflexion of the ankle in stance, the time of the minimum ab-adduction of the hip and the time of the minimum rotation of the pelvis. According to what observed above, the risky condition was characterized by early timing of peaks and increased peak amplitude. These four parameters did not have significant inter-conditions differences neither for the risk nor the safe scenario (Table 2, parameters with light blue background).

**Figure 2:**
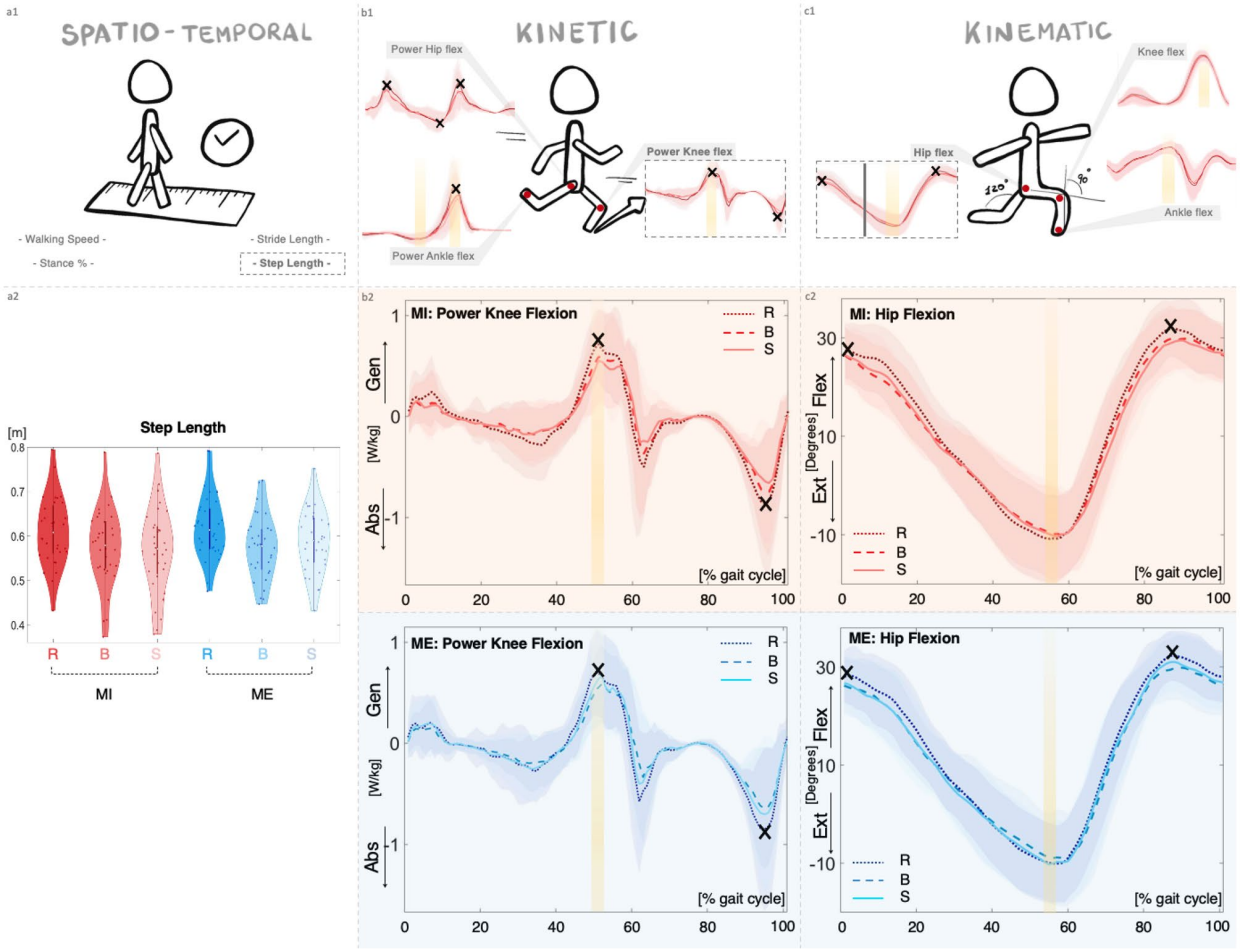
Graphical representation of parameters for which BR as well as SR are statistically significant for both MI and ME condition (corresponding to the parameters on white background in Table 1). [Top panel] [1]: Spatio-temporal parameters (left, [a1]), kinetics curves (middle, [b1]) and kinematics curves (right, [c1]) are reported. The “X” symbols highlight statistically significant differences in the amplitude of peaks, while the yellow bars refer to the statistically significant differences in the timing of peaks. Gray bar in [c1] indicates statistical significance for the range of motion (ROM) value. Curves illustratively refer to the MI condition (accordingly, they are depicted with red nuances. See below). Please, note that the illustration depicts a specific subset of parameters satisfying the “risky-scenario-driven” approach (i.e., BR as well as SR are statistically significant both for MI and ME, corresponding to the parameters on white background in Table 1). [Bottom panel, Left part] [a2]: Illustrative spatio-temporal data. Violin plot of the step length parameter for the MI (red) and ME (blue) condition; shade of red/blue are used for the safe (light), baseline (medium), and the risky (dark) scenarios. [Bottom panel, Middle part and Right part]: Illustrative kinetic data [b2]. Curves representing the power of the knee during flexo-extension in the MI condition (up), and in the ME condition (down). Illustrative kinematic data [c2]. Curves representing the hip flexo-extension in MI condition (up), and in the ME condition (down). For both kinetic and kinematic illustrative data, each curve represents the median curve considering 32 participants. Distinct “colors + type of line” combinations refer to the distinct conditions and scenarios (for MI: dark red + dots = risky; medium red + dashed line = baseline; light red + line = safe) (for ME: dark blue + dots = risky; medium blue + dashed line = baseline; light blue + line = safe). The areas in the background are the interquartile ranges. The “X” symbols highlight statistically significant differences in the amplitude of peaks, while the yellow bars refer to the statistically significant differences in the timing of peaks. *MI:* Motor Interference condition, *ME:* Motor Expectation condition, *B:* Baseline, *R:* Risky scenario, *S:* Safe scenario.

To summarize our results, Fig. 2 synthesizes the “risky-scenario-driven” approach (i.e., significant results for BR as well as SR, in MI and/or ME condition). Notably, one illustrative spatio-temporal parameter (i.e., step length), one kinetic (i.e., power of the knee in flexion), and one kinematic feature (i.e., hip flexion) for the risky/safe scenarios and the baseline are reported for both conditions (MI and ME).

### Machine learning analysis

The ML classifier ability to discriminate between risky and safe scenarios is reported in Table 3, considering both pooled and unpacked (i.e., spatio-temporal, kinetic, and kinematic) subsets of data.

**Table 3:** Performance metrics (Accuracy, AUC, and F1-score) for the pooled and unpacked (i.e., spatio-temporal; kinetic; kinematic) subsets of parameters. For each performance metric index, the p-value computed using a permutation test resulted statistically significant (p < 0.05). *AUC:* Area under the curve.

Parameters	Classification performances		
	Accuracy	AUC	F1-score
	62.9%	62.9%	62.7%
	64.8%	64.8%	63.5%
	65.6%	65.6%	64.9%
	54.3%	54.3%	56.9%

Considering the pooled set of parameters, the linear classifier significantly discriminated between risky and safe scenario with 62.9%-Accuracy, 62.9%-AUC, and 62.7%-F1 score (all p-values < 0.009). Unpacking the parameters on the basis of the type of measure into more specific subsets, the linear classifier still significantly discriminated between risky and safe scenario in all three cases (i.e., spatio-temporal, kinetic, and kinematic). However, performances were different in the distinct subsets of data. Kinetic parameters led to the best classification performance for each index, also overcoming the performance indices obtained in the pooled case. Spatio-temporal parameters also scored high performance metrics, similar to kinetic subset of data, and also higher than the pooled set of parameters. Differently, kinematic parameters were characterized by the lowest classification performance metrics. It suggests that even if they still significantly discriminate between the risky versus safe scenario, their discriminative information content is noisier and already included in the spatio-temporal/kinetic subsets of data.

To maximize the reliability of the SVM approach, all these results were obtained by merging MI and ME condition sets of data. For sake of completeness ML classifier performances for both conditions taken separately (MI and ME) can be found in the supplementary material section (Supplementary Tables S3 and S4, respectively).

## Discussion

For centuries the motor system was considered the unique “actor” driving walking activities as well as any movements. This classical view assumed that motor areas were demanded to control and execute actions, whereas no “higher” (i.e., cognitive) contribution was hypothesized in motor performance. More recently, this view has been progressively overcome. Accordingly, multiple versions of tasks testing cognitive functioning during walking have been provided (i.e., dual-task experimental paradigm). Among them, cognitive tasks included talking, memory recall, and arithmetic calculation^26–30^. All these studies clearly ascertained an impact of cognitive effort on walking pattern, and this assumed clinical relevance in monitoring both motor and neurocognitive functioning (e.g., in ageing). An additional support to unpack distinct computations supporting walking activity was indirectly provided by studies focused on action-execution and action-observation matching^31,32^. These mechanisms basically assumed that the brain recruits very similar neural circuits both when one executes an action (e.g., to grasp a bottle for drinking), and when she/he observes the same action performed by another individual. From a neurophysiological perspective, this indicates that the brain encodes the “motor representation” of specific actions, regardless they are in first-person executed or simply observed^33^. From a more theoretical perspective, this also means that the rigid separation between the motor system that executes actions and the sensory system that perceives others’ actions should be overcome^34^. Noteworthy, “motor representation” shows astonishing properties of *generalization*^35,36^, *abstraction*^37,38^, and *socially-oriented-tuning*^39–41^ that fit well also with the idea that we actively collect (rather passively register) sensory information^13,16,42^. To summarize, these lines of research opened further intriguing insights into the understanding of high-level, flexible, non-motor computations that play a role in driving motor representation, and in turn any motor performances (including walking activity). However, they did not directly tackle an additional critical point, that is the individual’s predictions. It is exactly what we tried to do in this work testing the impact of distinct scenarios expectation on walking pattern.

In this study, we proposed a specific focus on sensory and perceptual information that can influence motor representation, and in turn motor execution, of walking. Concerning motor interference, our results confirm and also extend previous findings described in the literature. Participants’ locomotion speed was reported to be slower when walking activity on asphalt is artificially paired with footstep-simulated sounds of different surface materials. Notably, surface material differences in terms of compliance explained walking speed variation (simulated surfaces with lower compliance than asphalt, e.g. snow or gravel, reduced walking speed) (Ref.^17^; see also Ref.^43^ for a preliminary rehabilitative application in lower-limb amputees). Our study extends these findings showing a more coherent and pervasive effect not only on spatio-temporal, but also on kinetic, and kinematic parameters. However, the key result of this study concerns the impact of priors on walking pattern. Our main aim was to demonstrate that simple auditory stimulation (i.e., audio risky/audio safe) is enough to influence walking pattern not only when the auditory stimulation is concurrent to the walking activity (motor interference), but also when the auditory stimulation served as prior to elicit specific expectations (motor expectation). This is exactly what we found for the risky scenario. The audio recalling a seaside during a flashing autumnal storm presented just before participants took the first step is enough to modify their gait pattern. As largely expected, data on motor interference was somehow more widespread and involved a larger number of gait features, and it was also consistent with participants’ explicit report at the end of the experimental session. While 17/32 participants reported at least a certain degree of awareness of the impact of auditory stimulation concurrently with the walking activity (motor interference), only 8/32 reported such an awareness for the stimulation occurred before the walking activity (motor expectation). Beyond it, our results clearly presented remarkable coherence between motor interference and motor expectation conditions. This suggests that our simple manipulation using auditory stimulation as prior is enough to generate a quantifiable effect comparable to the one produced by the online interfering auditory cues. Noteworthy, this effect is noticeable in a large set of parameters showing statistically significant inter-scenarios differences when the risky scenario is compared to the baseline (BR) as well as to the safe scenario (SR). The fact that these parameters simultaneously showed non-significant inter-conditions differences in the risky scenario speaks in favor of an effect primarily driven by the scenario (and not by the condition). Specifically, participants increased their walking speed, their step and stride length, and reduced the stance phase in the risky scenario. Furthermore, the risky scenario brought forward peculiar peaks of the gait kinematics and kinetics, among which the maximum flexion of the ankle and knee, and the generated power at the ankle. The increase of the movement amplitude at proximal level (i.e., the hip), and the power at the joints also characterized the participants’ gait both in motor interference and motor expectation conditions. Noteworthy, participants were requested to walk on the treadmill in self-paced modality. This not only excludes that they may be forced to modify their walking pattern by “unnatural” walking speed, but also “normalizes” for a sort of individual walking speed threshold. Unsurprising considering the participants’ characteristics, our findings seem to indicate that the safe scenario basically did not differ- or to the utmost differs very marginally—from the baseline.

Interestingly, from a different but convergent level of analysis also our machine learning approach supports the main results reported above. We applied an SVM classifier to explore the possibility that our scenarios (risky versus safe) can be decoded from our pooled (spatio-temporal + kinetic + kinematic) or unpacked (i.e., spatio-temporal or kinetic or kinematic) subset of parameters. Using the pooled set of parameters, the linear classifier significantly discriminates between risky and safe scenario. The unpacked approach aimed to better elucidate the specific contribution of each subset of data, and also spatio-temporal/kinetic/kinematic based classifiers significantly discriminated between the two scenarios. Our results indicate that kinetic and spatio-temporal parameters show the best classification performance for each index, even overcoming the performance indices obtained in the pooled combination. In contrast, results using the kinematic subset of data suggest that even if the model significantly discriminates the risky versus safe scenario, its information content is noisier and likely already included in the spatio-temporal/kinetic subsets of data.

Although further data and additional experiments should be provided to draw more comprehensive conclusions, our study may assume strong theoretical and clinical relevance. What we expect to hear or see interferes with, and even supersedes, what we actually hear and see. Accordingly, to interpret sensory information entails not only the weighting of incoming sensory evidence, but also its balancing with preexisting knowledge (priors). Our brain basically tries to promote the most efficient encoding of highly recurrent events and, in parallel, to minimize information missing^42,44^. Thus, having expectations about the world is a tool to anticipate the future and, in turn, to promote more fluent interactions with objects and people facilitating plausible interpretations from noisy and ambiguous data^45^. To the best of our knowledge, no studies in the literature have specifically and systematically explored this aspect in reference neither to lower nor to upper limb activity using well-controlled experimental designs and state-of-art motion capture techniques. The only findings barely addressing this topic were generally not able to disentangle the “pure” effect of expectations from the “real” effect due to treadmill perturbations^46^. In contrast, an interesting work may provide indirect support to our findings^47^. Using psychophysics methods, and benefiting from a rigorous motion capture dataset previously recorded^48^, the authors tried to explore how prior expectations concerning others’ intentions were integrated with observed motion pattern. Participants were requested to discriminate videos concerning distinct types of action (grasp-to-pour vs. grasp-to-drink) after having received congruent/incongruent cues. Noteworthy, certain videos were “transparent” (i.e., participants were accurate in discriminating them when they have access exclusively to motion pattern), whereas other ones were “opaquer” (i.e., participants committed a larger number of errors in discriminating them when they have access exclusively to motion pattern). Using drift diffusion model, Koul et al.^47^ demonstrated that specific motion features predicted the observers’ intention choice in case of “transparent” stimuli, whereas prior expectations (i.e., congruent/incongruent cue) predicted observers’ intention choice in case of “opaquer” stimuli. These findings provide support for a layered intention decoding processing in which distinct elements (motion patterns, priors) were differently weighted according to the (motion patterns) informativeness of the stimuli. Thus, this can be considered—at least indirectly—in agreement with our hypothesis assuming an impact of expectations also on gait patterns (see also Ref.^49^).

From a clinical perspective, our study may assume significance in the context of aging-related conditions, as also sketched in the “Introduction” section^5^. Indeed, being both reduced gait proficiency and general cognitive decline well-establishing both in non-clinical and clinical ageing, our study may represent an alternative experimental approach for testing these aspects. Noteworthy, the framework emerging from our results may provide hints for a deeper understanding of both cerebellar related disturbances, and neurodevelopmental conditions such as Autism Spectrum Disorder (ASD). In general terms, both gait pattern disturbances and predictive coding anomalies have been reported in these clinical conditions^50–53^. In addition, the cerebellum has been implicated in the pathophysiology of ASD^54^, and autistic-like behaviors have been reported in cerebellar related disturbances^4^. However, such general references do not seem to have neither strong operational impact nor clear role in understanding neurocomputational peculiarities of these clinical conditions. Probably, we should promote a refined, and more sophisticated approach to non-motor computations impacting on motor functioning. If the cerebellum plays a role in weighting predictions and expectations^55–57^, and gait disturbances are widely ascertained in patients with cerebellar related disturbances^10,58^, then a deeper understanding of distinct non-motor computations involved in cerebellar related disturbances may be pivotal to address personalized rehabilitative approaches addressing walking proficiency. Concerning ASD, a particular focus has been recently provided to disentangle potential anomalies in weighting sensory information during updating of the probabilistic representation of the environment^52^. In other words, it has been hypothesized that incoming sensory signals are weighted anomalously when integrated with the brain’s existing model of the environment, in turn impacting the use that individuals with ASD do of this body of information. If—and eventually how—such a different way of weighting information may lead to significant behavioral effects (e.g., in social interactions) remains matter of vibrant debate^59–61^. More specifically, if—and eventually how—anomalies in using priors may impact upper or lower limb activity (e.g., does anomalous proprioceptive feedback of the terrain impact the planning of following steps ?) remains largely unexplored, although in the last decade a generic reference to motor symptoms in ASD was often reported by clinicians (Ref.^50^, but see also Ref.^62^).

Finally, the framework emerging from our results may provide strong significance also in neuroprosthetics, and in general in research adopting advanced technological solutions (i.e., neurotechnologies, see Ref.^63^). For example, active exoskeleton and brain-computer interface methodologies continuously deal with technical, technological, and theoretical challenges. Intention decoding (e.g., to start walking vs. to stop walking; to start walking on the grass vs. to start walking on the asphalt) represents one of the most urgent challenges in this domain^64^, being strictly related to safety and usability of these neurotechnologies^43,63,65^. Our study suggests that priors and expectations should be also considered in the complex picture composing the intention decoding issue for walking. Even if it is not directly applicable to our experimental design considering the absence of any visual stimulation, it is noteworthy the reference to a couple of innovative eye-tracking studies suggesting that we can “map” our confidence about the terrain monitoring gazing activity^66,67^. How much such a confidence is influenced by priors and expectations, and how priors are not set in stone but rather reflect the individual (and fluctuating) model of the world, represent further fascinating challenges.

### Limitations of the study

Our study provides direct, combined, and convergent evidence supporting the role of prior and expectations in modifying walking patterns in healthy young adults. However, as largely discussed, our main effects refer to the risky scenario. In contrast, motion patterns in the safe scenario barely coincided with the ones during the baseline. We hypothesized that it was essentially due to the participants’ characteristics (healthy young adults). A critical future challenge would be the testing of older people or individuals with clinical conditions impacting on gait proficiency. An additional limitation may concern the absence of signals to be coupled with the motion analysis. For example, the presence of wearable electroencephalography (EEG) system recording brain rhythms during walking may provide further insights on the predictive processing via neural decoding of gait phases^65,68^. Its absence in our study does not permit to directly demonstrate anything at the neural level. A further possibility may regard the use of eye tracking systems. Combining eye tracking and motion capture may not only definitely exclude any distraction effects (indeed very unlikely in our setup considering the participants’ characteristics and the dimly illuminated room). More intriguingly, gaze control may offer convergent insights on participants’ visuo-motor coupling, as recently suggested in the literature, and briefly sketched in the “Discussion” section^11,66,67^.

The main limitation of this work may appear the absence of interpretation at the level of single parameter variation. However, it is likely a more general constraint of all motion capture approaches that normally rely on combined/multiple parameters interpretation of data. This is primarily due to the fact that single parameter variation becomes more and more reliable when it is considered taking into account the “global” pattern variation, i.e., when single parameter is considered in “synergy” with other ones. Noteworthy, a similar approach is also employed for the study of patterns of muscles activations with electromyography (EMG) (i.e., “motor synergies”, see Refs.^3,69,70^). According to such a general perspective, our results grouped into specific sub-clusters (spatio-temporal; kinetic; kinematic) appear largely consistent with findings reported in the literature^71^. A further limitation may concern the fact that we did not control for potential rhythmic/cyclic features of the audios (risky, safe), that may impact on gait pattern and consequently bias our findings. Although a priori reasonable, it is very unlike that rhythmic/cyclic auditory patterns in the audios (e.g., sea waves) play a relevant role in our results. Indeed, taking for example the waves of our audios, a simple qualitative analysis clearly ascertains “irregular” patterns (as if they were waves near cliffs, and not “regular” waves on the water’s edge). In addition, waves sounds were always mixed with other background sounds (e.g., cliff seagull sounds).

Finally, two points deserve some additional attention. Our work refers to a specific risky (safe) scenario, but we cannot generalize our results for whatever risky (safe) scenario. This means that—for example—we can hypothesize a risky scenario in which participant’s mean velocity is reduced (e.g., risk to fall on the ice), and another one in which mean velocity is increased (e.g., risk to miss your train). Although certain scenarios seem to be unequivocal, other ones may be biased by individual traits, personal preferences, and attitudes (e.g., barking of a dog). Considering that our principal aim was to demonstrate the possibility that even a simple auditory stimulation used as prior may affect “pure” walking pattern (i.e., the condition we referred to as Motor Expectation), our work does not permit to explore in deep such a point. Future studies should combine psychological and motor neuroscience approaches to disentangle such an aspect. Last, we found a certain number of parameters that fulfilled the so-called “risky-scenario-driven” approach only in the MI or ME condition (pink background and light blue background in the tables, respectively). From the one side, this may be simply due to slight fluctuations of significance levels that may occur in motion capture or other experimental approaches when—for practical reasons—sample sizes are reasonably good (but not optimal). From the other and more interesting side, these differences between MI and ME conditions may effectively reflect the distinct basic processing of interference and expectation computations. It is totally reasonable, and also consistent with the literature, that the processing of a concurrent and a preceding stimulus are mediated by partially overlapping—but not completely identical—brain computations. Although this hypothesis opens intriguing insights for additional considerations, we think it remains—at least in the context of our study—largely speculative. Any interpretation would be an over-interpretation. Future studies will have to challenge this important point with ad hoc experimental designs.

## Conclusion

Our study offers direct, combined, and converging evidence concerning the role of priors and expectations in modifying walking patterns. Eliciting a “risky and potentially dangerous” scenario through ad hoc audio-descriptions, we provided evidence that participants’ walking patterns were impacted not only by concurrent stimulation (motor interference), but also by preceding stimulation served as prior (motor expectation). Noteworthy, our results indicated remarkable coherence among spatio-temporal, kinetic, and kinematic pattern modifications in both conditions (MI, ME), as synergistically proved by convergent statistical and machine learning approaches. This raises intriguing research and clinical hints for a deeper understanding of the interactions between motor performance, sensory/perceptual processing, and the use of past information or knowledge (predictive coding).

## Supplementary Information

Supplementary Information.

## Data Availability

Data and code will be deposited at the Zenodo repository. Further information should be directed and will be fulfilled by the lead contact, Emilia Biffi [emilia.biffi@lanostrafamiglia.it].
