# Supplementary material for: Context expectation influences the gait pattern biomechanics
Source: Sci Rep. 2023 Apr 6;13:5644. doi: 10.1038/s41598-023-32665-7 (PMC10079826; doi:10.1038/s41598-023-32665-7)
Supplement: Supplementary file 1 — Supplementary Information. [file 41598_2023_32665_MOESM1_ESM.pdf]

Context expectation influences the gait pattern biomechanics

Ciceri Tommaso, Malerba Giorgia, Gatti Alice, Diella Eleonora, Peruzzo Denis, Biffi Emilia*, Casartelli Luca.

Correspondence to:

Emilia Biffi, PhD
Scientific Institute IRCCS E.Medea
Bioengineering Lab
Bosisio Parini, Lecco (Italy)
Email: emilia.biffi [@] lanostrafamiglia [.] it

			MI R	MI B	MI S	ME R	ME B	ME S	p-value MI BR	p-value MI BS	p-value MI SR	p-value ME BR	p-value ME BS	p-value ME SR
Spatio-temporal		Step Width [cm]	12.4(3.9)	12.3(3.7)	12.0(2.9)	12.2(3.6)	12.2(3.6)	12.1(3.8)	-	-	-	0.0025	< 0.001	-
Kinematic	KNEE	ROM Flex [°]	65.3(5.8)	65.3(7.5)	65.0(8.0)	65.9(5.0)	64.9(6.4)	65.5(5.6)	-	-	-	< 0.001	0.0039	-
	HIP	Max AbAd [°]	9.9(4.3)	9.2(4.3)	9.2(4.5)	9.9(4.1)	8.6(3.7)	9.2(4.1)	0.0021	0.0014	-	< 0.001	0.0020	-
		Mean Rot-stance [°]	5.0(5.0)	4.6(4.2)	5.6(5.7)	4.9(4.2)	5.3(4.4)	5.1(4.7)	-	-	-	0.0052	-	-
		Min AbAd [°]	-9.3(3.6)	-8.9(4.4)	-9.9(4.5)	-9.9(5.0)	-9.7(4.6)	-10.2(4.3)	-	0.0062	-	0.0077	-	-
		Min Flex [°]	-11.3(8.2)	-10.7(9.0)	-11.3(9.1)	-11.1(7.4)	-10.1(9.7)	-11.0(8.0)	-	-	-	0.0013	-	-
		ROM AbAd [°]	18.7(5.7)	17.6(6.3)	18.4(7.7)	18.2(6.5)	17.7(6.6)	18.3(6.8)	0.0027	0.0013		< 0.001	0.0039	-
		T Max AbAd [%]	20.1(3.5)	20.8(4.3)	21.1(4.6)	20.2(3.2)	21.1(3.6)	20.8(4.2)	-	-	-	0.0139	-	-
	PELVIC	Mean Rot [°]	-0.2(2.2)	-0.0(2.5)	0.1(2.0)	-0.2(2.0)	-0.3(2.1)	0.1(2.4)	-	-	-	-	0.0147	-
		Min Obl [°]	-6.6(4.7)	5.6(4.4)	-5.5(4.5)	-6.8(4.3)	-5.7(3.8)	-6.7(4.3)	0.0125	-	-	< 0.001	-	-
		ROM Obl [°]	13.4(3.9)	12.3(4.1)	12.6(5.3)	13.7(4.4)	12.6(5.7)	12.9(5.1)	0.0017	-	-	< 0.001	0.0065	-
		Max Obl [°]	6.5(3.1)	6.1(2.6)	6.7(3.0)	7.1(3.1)	6.6(3.3)	7.1(2.7)	0.0034	0.0132	-	< 0.001	0.0113	-
	TRUNK	Max Flex [°]	7.9(4.0)	7.8(4.1)	7.7(4.9)	7.8(4.3)	6.3(4.8)	7.8(4.8)	-	-	-	< 0.001	-	-
		Min Flex [°]	-7.7(4.9)	-6.8(5.4)	-6.9(5.3)	-7.6(4.5)	-6.7(5.2)	-7.3(4.3)	< 0.001	0.0091	-	< 0.001	-	-
		ROM Tilt [°]	4.1(2.1)	3.9(2.3)	4.1(2.4)	4.3(1.7)	4.1(1.6)	4.0(1.7)	-	< 0.001	-	-	-	-
		ROM Rot [°]	11.5(5.4)	11.0(3.8)	10.9(4.8)	11.5(3.3)	10.8(3.9)	11.5(3.7)	-	-	-	< 0.001	-	-

Supplementary Table S1.

Results of the inter-scenarios analysis showing statistically significant results at the Friedman Test ($p < 0.05$), and referring to the parameters that *do not* satisfy the risky-scenario-driven approach requirements. Notably, they have not significant results - in MI and/or ME - at the post hoc test between the risky scenario *versus* the baseline (BR) as well as in the risky scenario *versus* the safe one (SR) (Wilcoxon Test with Bonferroni correction, with $p < 0.016$). Median and IQR values are reported.

[Legend]

MI: Motor Interference condition; ME: Motor Expectation condition;

B: Baseline; R: Risky scenario; S: Safe scenario; BR: Baseline Vs. Risky scenario; SR: Safe scenario Vs. Risky scenario; BS: Baseline vs Safe scenario.

			MI		ME		p-value MI vs ME	
			**MI_R**	**MI_S**	**ME_R**	**ME_S**	**MI_R vs ME_R**	**MI_S vs ME_S**
**Spatio-temporal**		Step Width [cm]	-0.3(1.0)	-0.3(0.8)	-0.6(1.5)	-0.6(0.8)	**0.042**	0.063
**Kinematic**	KNEE	ROM Knee Flex [°]	0.5(1.5)	-0.2(1.5)	1.6(3.1)	1.1(2.3)	0.169	**0.015**
	HIP	Max Hip AbAd [°]	0.4(0.9)	0.2(0.9)	0.5(1.0)	0.5(1.0)	0.200	0.903
		Mean Hip Rot-stance [°]	-0.3(1.0)	-0.0(1.0)	-0.4(0.9)	0.0(1.0)	0.087	0.091
		Min Hip AbAd [°]	-0.2(0.9)	-0.5(0.8)	-0.5(1.1)	-0.3(0.7)	0.758	0.274
		Min Hip Flex [°]	-0.4(1.6)	-0.3(1.3)	-0.7(0.9)	-0.1(1.8)	0.308	0.647
		ROM Hip AbAd [°]	0.8(1.5)	0.9(1.7)	1.1(1.8)	0.9(1.7)	0.266	0.633
		Time Max Hip AbAd [%]	-0.3(1.1)	0.1(1.7)	-0.6(1.2)	0.1(1.0)	**0.0425**	0.3082
	PELVIC	Mean Pelvic Rot [°]	0.1(0.5)	0.1(0.6)	0.3(0.6)	0.3(0.7)	0.158	0.385
		Min Pelvic Obl [°]	-0.2(0.6)	-0.2(1.0)	-0.5(0.7)	-0.3(0.8)	0.235	0.772
		ROM Pelvic Obl [°]	0.6(1.3)	0.3(1.5)	1.0(1.4)	1.0(1.6)	0.221	0.903
		Max Pelvic Obl [°]	0.4(0.7)	0.2(0.7)	0.5(0.8)	0.3(1.0)	0.416	0.888
	TRUNK	Max Trunk Flex [°]	0.2(1.0)	0.2(1.3)	0.8(1.2)	0.3(1.4)	0.175	0.758
		Min Trunk Flex [°]	-0.6(0.8)	-0.5(1.1)	-0.7(1.1)	-0.2(1.0)	0.543	0.405
		ROM Trunk Tilt [°]	0.2(0.8)	0.3(0.4)	-0.0(0.5)	0.0(0.6)	0.556	**0.015**
		ROM Trunk Rot [°]	1.0(1.5)	0.2(2.0)	0.9(1.3)	0.4(1.3)	0.933	0.483

Supplementary Table S2.

Results of the inter-conditions analysis considering the risky and safe scenarios detrended respect to the baseline. All parameters correspond to the ones reported in Supplementary Table S1 (i.e., the ones that *do not* satisfy the "risky-scenario-driven" approach requirements). Median and IQR values of the detrended parameters (see Equations 1 to 4 in the Data Analysis section) are reported. P-values refer to the Wilcoxon test between MI and ME (i.e., MI_R Vs. ME_R; MI_S Vs. ME_S). Statistically significant values are in bold.

[Legend]

MI: Motor Interference condition; ME: Motor Expectation condition; MI_S: Motor Interference Safe (detrended); MI_R: Motor Interference Risky (detrended); ME_S: Motor Expectation Safe (detrended); ME_R: Motor Expectation Risky (detrended).

PARAMETERS	CLASSIFICATION PERFORMANCES		
	Accuracy	AUC	F1-score
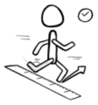 Pooled	64.1%	64.1%	66.9%
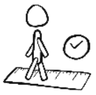 Spatio-temporal	65.6%	65.6%	62.4%
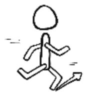 Kinetic	67.9 %	67.9%	63.8%
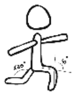 Kinematic	53.9%	53.9%	57.4%

Supplementary Table S3:
Performance metrics (Accuracy, AUC, and F1-score) for the pooled and unpacked (i.e., spatio-temporal; kinetic; kinematics) subsets of parameters in the MI condition. For each performance metric index, the p-value computed using a permutation test resulted statistically significant ($p < 0.05$).

[Legend]
AUC: Area Under the Curve; MI: Motor Interference condition

PARAMETERS	CLASSIFICATION PERFORMANCES		
	Accuracy	AUC	F1-score
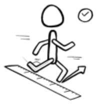 Pooled	66.4%	66.4%	62.2%
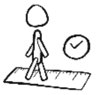 Spatio-temporal	60.9%	60.9%	57.5%
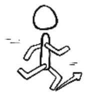 Kinetic	60.2 %	60.2%	60.2%
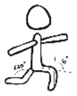 Kinematic	55.4%	55.4%	49.8%

Supplementary Table S4:
Performance metrics (Accuracy, AUC, and F1-score) for the pooled and unpacked (i.e., spatio-temporal; kinetic; kinematics) subsets of parameters in the ME condition. For each performance metric index, the p-value computed using a permutation test resulted statistically significant ($p < 0.05$).

[Legend]
AUC: Area Under the Curve; ME: Motor Expectation condition

ADDITIONAL MATERIALS

Text of the AUDIO-DESCRIPTION "Safe" (Italian)

* Siamo al mare in una spiaggia della Sardegna. E' una caldissima giornata di piena estate.

Spiagge dorate, cielo azzurro e limpidissimo. Non si vede una nuvola da settimane.

Luglio è probabilmente il mese più caldo dell'anno, e sono 18 giorni che la temperatura supera regolarmente i 35 gradi. Nemmeno l'ombra di una precipitazione o di una perturbazione; il mare è calmissimo, sembra disegnato; l'orizzonte mostra solo un maestoso, splendente e raggiante sole estivo.

Un comodo pontile in legno si staglia sulle limpide e cristalline acque, e camminando su di esso si può raggiungere facilmente una fantastica grotta marina.

Giovani, famiglie e bambini lo percorrono allegramente con in mano borse, secchielli e racchettoni...

Text of the AUDIO-DESCRIPTION "Risky" (Italian)

* Siamo al mare in una spiaggia della Sardegna. E' una cupa giornata di metà autunno.

Vento, pioggia battente e mare in burrasca. Il cielo è grigio, quasi sembra notte.

Solo la luce abbagliante dei lampi, accompagnata dal frastuono dei tuoni, illumina di tanto in tanto la spiaggia. Fa freddo, è umido, il mare è minaccioso e invade ripetutamente la spiaggia. Il rumore scrosciante delle onde si unisce a quello del vento, si sta infatti avvicinando una tromba d'aria.

Il pontile in legno sembra essere l'unica via possibile da percorre per raggiungere l'albergo e mettersi al riparo, sebbene sia continuamente sovrastato dall'impeto della mareggiata. E' totalmente bagnato, quasi non si vede a causa della forza del mare.

E non si vede anima viva nei paraggi, ma quella è l'unica via esistente per mettersi al riparo…
